# Syntheses and crystal structures of three [*M*(acac)_2_(TMEDA)] complexes (*M* = Mn, Fe and Zn)

**DOI:** 10.1107/S2056989019016372

**Published:** 2020-01-01

**Authors:** Jan Henrik Halz, Christian Heiser, Christoph Wagner, Kurt Merzweiler

**Affiliations:** a Martin-Luther-Universität Halle-Wittenberg, Naturwissenschaftliche Fakultät II, Institut für Chemie, D-06099 Halle, Germany

**Keywords:** crystal structure, acetyl­acetonate, tetra­methyl­ethylenedi­amine, transition metal complex

## Abstract

The complexes bis­(acetyl­acetonato-κ^2^ *O*,*O*′)(*N*,*N*,*N*′,*N*′-tetra­methyl­ethylenedi­amine-κ^2^ *N*,*N*′)manganese(II), [Mn(C_5_H_7_O_2_)_2_(C_6_H_16_N_2_)], bis­(acetyl­acetonato-κ^2^ *O*,*O*′)(*N*,*N*,*N*′,*N*′-tetra­methyl­ethylenedi­amine-κ^2^ *N*,*N*′)iron(II), [Fe(C_5_H_7_O_2_)_2_(C_6_H_16_N_2_)], and bis­(acetyl­acetonato-κ^2^ *O*,*O*′)(*N*,*N*,*N*′,*N*′-tetra­methyl­ethylenedi­amine-κ^2^ *N*,*N*′)zinc(II), [Zn(C_5_H_7_O_2_)_2_(C_6_H_16_N_2_)], were synthesized from the reaction of the corresponding metal acetyl­acetonates [*M*(acac)_2_(H_2_O)_2_] with *N*,*N*,*N*′,*N*′-tetra­methyl­ethylenedi­amine (TMEDA) in toluene. Each of the complexes displays a central metal atom which is nearly octa­hedrally surrounded by two chelating acac and one chelating TMEDA ligand, resulting in an N_2_O_4_ coordination set. Despite the chemical similarity of the complex units, the packing patterns for compounds **1**–**3** are different and thus the crystal structures are not isotypic.

## Abstract 2

Syntheses and crystal structures of three metal complexes [*M*(acac)_2_(TMEDA)] [*M* = Mn (**1**), Fe (**2**) and Zn (**3**)] with acetyl­acetonate and *N*,*N*,*N*′,*N*′-tetra­methyl­ethylenedi­amine are discussed.

## Chemical context   

Pentane-2,4-dionate (acac) and ethyl­enedi­amine derivatives are amongst the most widely used chelate ligands in transition metal chemistry. The crystal structures of mixed complexes [*M*(acac)_2_(TMEDA)] (TMEDA = *N*,*N*,*N*′,*N*′-tetra­methyl­ethylenedi­amine) containing both types of ligands have been reported for several divalent metals, *e.g. M* = V (Ma *et al.*, 1999 ▸), Co (Pasko *et al.*, 2004 ▸), Ni (Trimmel *et al.*, 2002 ▸; Zeller *et al.*, 2004 ▸) and Ru (Halbach *et al.*, 2012 ▸). The synthesis of [Zn(acac)_2_(TMEDA)] was reported recently in conjunction with the Ru derivative but without crystal structure determination (Halbach *et al.*, 2012 ▸). Typically, [*M*(acac)_2_(TMEDA)] complexes are used as valuable starting materials for the preparation of organometallic and coordination compounds (Kaschube *et al.* 1988 ▸; Nelkenbaum *et al.*, 2005 ▸; Albrecht *et al.*, 2019 ▸). Moreover, there is an increasing inter­est in [*M*(acac)_2_(TMEDA)] and related [*M*(hfa)_2_(TMEDA)] (hfa = 1,1,1,5,5,5-hexa­fluoro­pentane-2,4-dionate) complexes as precursor materials for CVD deposition of Co_3_O_4_ (Pasko *et al.*, 2004 ▸), Fe_2_O_3_ (Barreca *et al.*, 2012 ▸) and MnF_2_ (Malandrino *et al.*, 2012 ▸).
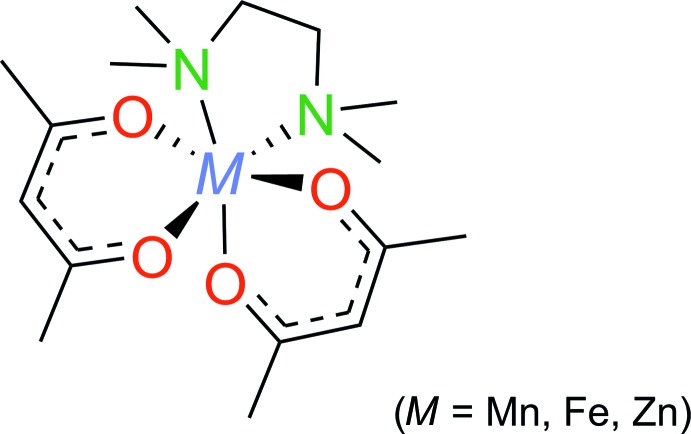


Typically, [*M*(acac)_2_(TMEDA)] complexes are synthesized from the reaction of the metal acetyl­acetonates with TMEDA. Following this procedure, we obtained the complexes [Mn(acac)_2_(TMEDA)] (**1**), [Fe(acac)_2_(TMEDA)] (**2**) and [Zn(acac)_2_(TMEDA)] (**3**) from the corresponding dihydrates [*M*(acac)_2_(H_2_O)_2_] and TMEDA in toluene as solvent. Recrystallization from *n*-hexane at 248 K afforded [Mn(acac)_2_(TMEDA)] (**1**) as yellow, [Fe(acac)_2_(TMEDA)] (**2**) as red–brown and [Zn(acac)_2_(TMEDA)] (**3**) as colorless products. Determination of the magnetic moments for [Mn(acac)**_2_**(TMEDA)] (5.7 B.M.) and [Fe(acac)_2_(TMEDA)] (5.1 B.M.) indicates a high-spin configuration in both cases.

## Structural commentary   

**Figure 1:**
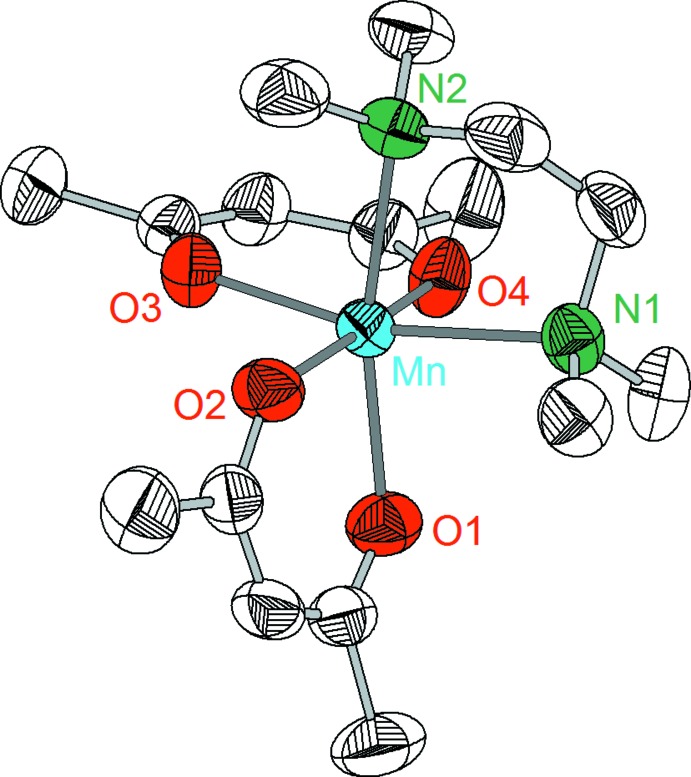
Mol­ecular structure of complex **1** showing the labeling scheme. Displacement ellipsoids drawn at 50% probability level, H atoms are omitted.

Compounds **1**–**3** crystallize in the monoclinic system, space group *P*2_1_/*n* with *Z* = 4. However, despite the similarity of the lattice parameters and the analogous mol­ecular structures, complexes **1**–**3** are not isotypic. The crystal structures consist of discrete complex mol­ecules [*M*(acac)_2_TMEDA] in which the central metal atoms are coordinated nearly octa­hedrally by four oxygen atoms of two acac ligands and two nitro­gen atoms of the TMEDA ligand (Figs. 1 ▸–3 ▸ ▸). Mn complex **1** exhibits Mn—O and Mn—N distances of 2.127 (1)–2.150 (1) Å and 2.356 (2)–2.364 (2) Å, respectively (Table 1 ▸). Similar geometric parameters have been reported for [Mn(acac)_2_(H_2_O)_2_] [Mn—O: 2.123 (8)–2.142 (8) Å; Mont­gom­ery & Lingafelter, 1968 ▸], [Mn(acac)_2_(1,10-phenanthroline)] [Mn—O: 2.116 (5)–2.152 (5) Å, Mn—N: 2.307 (5) Å; Stephens, 1977 ▸], [Mn(acac)_2_(2,2′-bi­pyridine)] [Mn—O: 2.148 (2)–2.158 (2) Å, Mn—N: 2.283 (2)–2.288 (3) Å; van Gorkum *et al.*, 2005 ▸] or [Mn(hfa)_2_(TMEDA)] [Mn—O: 2.139 (4)–2.178 (4) Å, Mn—N: 2.299 (5)—2.307 (5) Å; Mal­an­drino *et al.*, 2012 ▸].

The Fe—O and Fe—N distances in compound **2** [2.050 (1)–2.097 (1) Å and 2.302 (1)–2.318 (1) Å, respectively; Table 2 ▸] are on average shorter than the corresponding Mn—O and Mn—N distances in complex **1**. The Fe—O and Fe—N distances compare well with the data that have been observed in the compounds [Fe(acac)_2_(H_2_O)_2_] [Fe—O: 2.034–2.041 Å; Tsodikov *et al.*, 1995 ▸], [Fe(hfa)_2_(picoline)_2_] [Fe—O: 2.057 (1) Å, Fe—N: 2.190 (3)–2.224 (3) Å; Novitchi *et al.*, 2017 ▸] or [Fe(hfa)_2_(TMEDA)] [Fe—O: 2.064 (1)–2.094 (1), Fe—N: 2.229 (2) Å; Dickman *et al.*, 1998 ▸].

[Zn(acac)_2_(TMEDA)] (**3**) displays Zn—O and Zn—N distances of 2.061 (1)–2.077 (1) and 2.253 (1)–2.272 (1) Å, respectively (Table 3 ▸). In comparison with the iron complex **2**, the average metal–oxygen distances and metal–nitro­gen distances are slightly shortened. On the whole, the Zn—O and Zn—N distances in compound **3** are similar to those observed in the related compounds [Zn(acac)_2_(H_2_O)_2_] [Zn—O: 2.032 (1)–2.049 (1) Å; Harbach *et al.*, 2003 ▸], [Zn(acac)_2_(1,10-phenanthroline)] [Zn—O: 2.044 (1)–2.085 (1) Å, Zn—N: 2.196 (1) Å; Brahma *et al.*, 2008 ▸], [Zn(acac)_2_(2,2′-bi­pyridine)] [Zn—O: 2.051 (1)–2.089 (1) Å, Zn—N: 2.197 (2)–2.208 (2) Å; Brahma *et al.*, 2008 ▸] or [Zn(hfa)_2_(TMEDA)] [Zn—O: 2.103 (1)–2.126 (1) Å, Zn—N: 2.145 (1)–2.151 (1) Å; Ni *et al.*, 2005 ▸].

In general, the above-mentioned [*M*(hfa)_2_(TMEDA)] (*M* = Mn, Fe, Zn) complexes exhibit shorter *M*—N distances than the corresponding [*M*(acac)_2_(TMEDA)] complexes. This effect is probably due to the electron-withdrawing effect of the CF_3_ groups of the hfa ligands.

The iron complex **2** displays a subtle elongation (0.041 Å) of the Fe—O bonds *trans* to the N atoms with respect to the Fe—O bonds *trans* to oxygen. A similar effect was observed for [Co(acac)_2_(TMEDA)] (Pasko *et al.*, 2004 ▸). In the case of the Mn and Zn complexes **1** and **3**, the *trans* influence is negligible as reported for [Ni(acac)_2_(TMEDA)] (Trimmel *et al.*, 2002 ▸) and [Ru(acac)_2_(TMEDA)] (Halbach *et al.*, 2012 ▸). A reverse effect with a shortening of the Zn—O bonds *trans* to nitro­gen was detected for [Zn(acac)_2_(2,2′-bi­pyridine)] and [Zn(acac)_2_(1,10-phenanthroline)] (Brahma *et al.*, 2008 ▸).

Each of the complexes **1**–**3** exhibits nearly planar six-membered acac-*M* chelate rings. The maximum deviation from planarity, as indicated by the dihedral angle between the M/O1/O2 (M/O3/O4) plane of the chelate ring and the best plane through O1/C2/C3/C4/O2 (O3/C7/C8/C9/O4), is 6.2 (1)° in the case of the zinc complex **3**. *PLATON* (Spek, 2009 ▸) was used to calculate the dihedral angles. The five-membered *M*-TMEDA ring adopts a twist conformation with approximate *C*_2_ symmetry. As a result of the centrosymmetric crystal structure, both types of the enanti­omeric chelate rings with λ and δ conformations are present.

The *M*O_4_N_2_ coordination polyhedra in compounds **1**–**3** deviate moderately from a regular octa­hedron. The O—*M*—O angles are in the range 171.7 (1)° (complex **1**) to 175.2 (1)° (complex **3**) and the N—*M–*-O angles vary from 161.3 (1)° (complex **1**) to 170.9 (1)° (complex **2**). The smallest acac bite angle is observed in compound **1** [83.6 (1)°], the largest is found in compound **3** [88.0 (1)°]. In the case of the TMEDA ligands, the bite angles are marginally smaller with a range between 77.3 (1)° (compound **1**) and 80.3 (1)° (compound **3**). Overall, the distortion of the *M*O_4_N_2_ octa­hedra in compounds **1**–**3** is very similar to that observed in the analogous V, Ni and Co complexes [*M*(acac)_2_(TMEDA)].

## Supra­molecular features   

**Figure 4:**
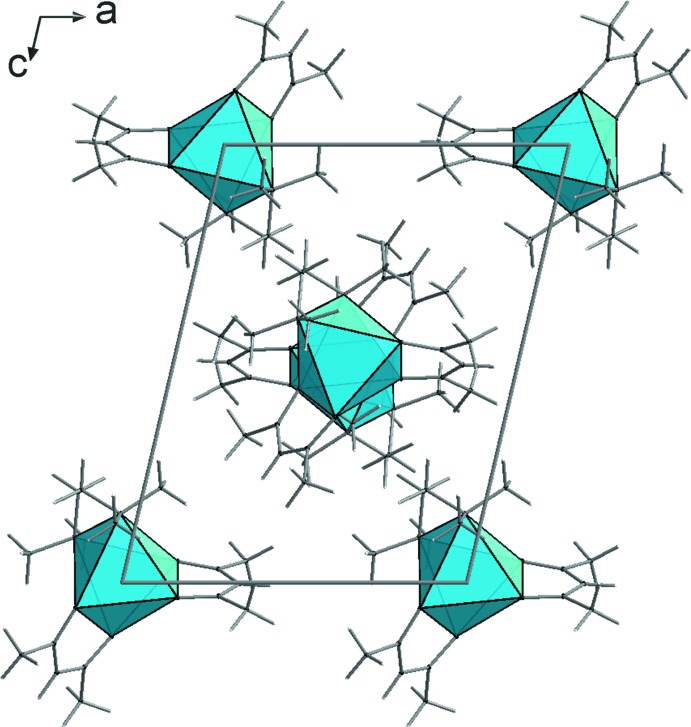
Crystal structure of compound **1**, viewed along the *b* axis.

**Figure 5:**
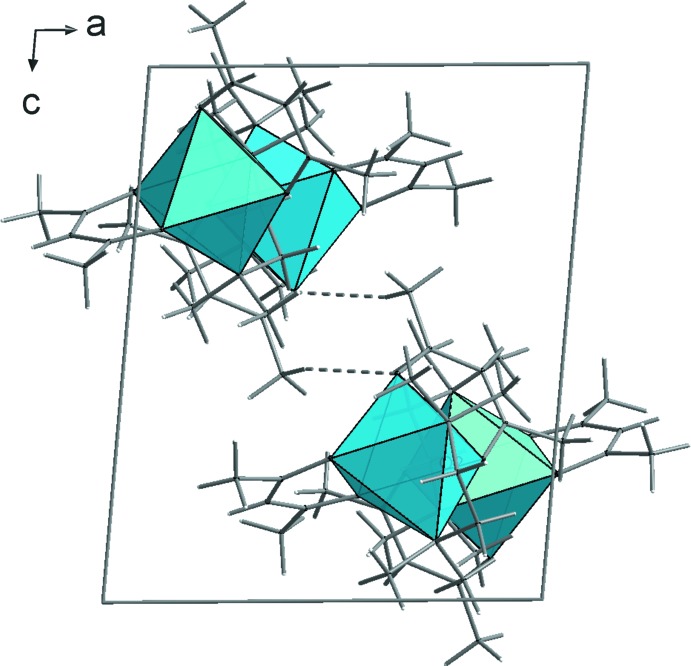
Crystal structure of compound **2**, viewed along the *b* axis. The inter­molecular C—H⋯O hydrogen bonds are shown as dashed lines.

The packing of the [*M*(acac)_2_(TMEDA)] units is dominated by van der Waals inter­actions. The mutual arrangement of the complex units **1**–**3** is similar but not identical (Figs. 4 ▸–6 ▸ ▸). In the case of the iron compound **2** there is also a contribution from weak C—H⋯O hydrogen bridges (Table 4 ▸). As a result, the complexes are associated by *R*_2_^2^(8) type motifs, forming centrosymmetric dimers (Fig. 5 ▸).

## Database survey   

A search in the Cambridge Structural Database (CSD, Version 5.40, February 2019 update; Groom *et al.*, 2016 ▸) for complexes with a composition [*M*(acac)_2_(TMEDA)] analogous to **1**–**3** revealed the crystal structures for the *M* = V, Ni, Co and Ru derivatives (Ma *et al.*, 1999 ▸; Pasko *et al.*, 2004 ▸; Trimmel *et al.*, 2002 ▸; Zeller *et al.*, 2004 ▸; Halbach *et al.*, 2012 ▸). However, none of these complexes is isotypic with the three title compounds. In the case of the related hfa derivatives, complexes of the type [*M*(hfa)_2_(TMEDA)] (hfa = 1,1,1,5,5,5-hexa­fluoro­pentane-2,4-dionate) with *M* = Mg, Mn, Fe, Co, Cu and Zn have been reported.

## Synthesis and crystallization   

TMEDA (7.5 ml, 5.8 g, 50 mmol) was added to a suspension of [*M*(acac)_2_(H_2_O)_2_] (25 mmol, *M* = Mn: 9.71 g, Fe: 9.73 g, Zn: 9.97 g) in toluene (30 ml). The suspension was stirred at 323 K for 2 h. After removal of the solvent under reduced pressure, *n*-hexane (25 ml) was added and insoluble parts were filtered off. The filtrates were kept at 248 K to obtain the products as yellow (**1**), red–brown (**2**) and colourless (**3**) crystalline solids in yields around 90%.

**Characterization**

[Mn(acac)_2_TMEDA] (**1**)

C_16_H_30_MnN_2_O_4_ calculated C 52.03, H 8.19, N 7.59%, found: C 51.71, H 8.13, N 7.14%; IR (ATR): ν = 3067 *w*, 2993 *w*, 2970 *w*, 2917 *w*, 2986 *w*, 2860 *w*, 2828 *w*, 2788 *w*, 2772 *w*, 1595 *m*, 1512 *s*, 1468 *m*, 1449 *m*, 1412 *s*, 1391 *m*, 1353 *m*, 1288 *m*, 1251 *m*, 1190 *w*, 1159 *w*, 1124 *w*, 1095 *w*, 1063 *w*, 1045 *m*, 1026 *w*, 1011 *m*, 950 *m*, 934 *w*, 913 *m*, 794 *m*, 771 *w*, 751 *m*, 650 *w*, 583 *w*, 526 *m*, 468 *w*, 448 *w*, 436 *w*, 400 *s*, 325 m, 212 s cm^−1^.

M.p.: 362 K.

[Fe(acac)_2_TMEDA] (**2**)

C_16_H_30_FeN_2_O_4_ calculated C 51.90, H 8.17, N 7.57%, found: C 51.75, H 8.08, N 7.23%; IR (ATR): ν = 3074 *w*, 3001 *w*, 2967 *w*, 2911 *w*, 2869 *w*, 2836 *w*, 2790 *w*, 1583 *m*, 1510 *s*, 1455 *m*, 1411 *s*, 1382 *m*, 1357 *w*, 1289 *m*, 1274 *w*, 1256 *m*, 1188 *w*, 1165 *w*, 1127 *w*, 1101 *w*, 1030 *w* 1012 *m*, 952 *m*, 917 *m*, 793 *m*, 762 *s*, 651 *w*, 583 *w*, 543 *m*, 475 *w*, 436 *w*, 404 *w*, 382 *s*, 296 *w*, 265 *m*, 227 *s* cm^−1^.

M.p.: 361 K.

[Zn(acac)_2_TMEDA] (**3**)

C_16_H_30_N_2_O_4_Zn calculated C 50.60, H 7.96, N 7.38%, found: C 50.33, H 8.13, N 7.23%; ^1^H-NMR (CDCl_3_, 399.962 MHz) δ = 5.15 [*s*, 2H, C(O)C*H*C(O)], 2.49 (*s*, 4H, Me_2_N-C*H*_2_), 2.31 (*s*, 12H, (C*H*_3_)_2_N), 1.85 [*s*, 12H, C*H*_3_C(O)]; ^13^C-NMR (CDCl_3_,100.581 MHz) δ = 190.9 [*C*(O)], 98.4 [C(O)*C*HC(O)], 56.5 (N*C*H_2_), 46.6 [(*C*H_3_)_2_N], 28.3 (C(O)*C*H_3_) ppm; IR (ATR): ν = 3071 *w*, 3001 *w*, 2975 *w*, 2881 *w*, 2835 *w*, 2792 *w*, 1615 *m*, 1593 *m*, 1515 *s*, 1469 *m*, 1455 *m*, 1411 *m*, 1390 *s*, 1354 *m*, 1290 *m*, 1252 *m*, 1190 *w*, 1166 *w*, 1128 *w*, 1101 *w*, 1061 *w*, 1032 *m*, 1013 *s*, 953 *m*, 936 *w*, 918 *m*, 798 *m*, 770 *m*, 754 *m*, 649 *w*, 584 *w*, 543 *m*, 474 *w*, 440 *m*, 405 *s*, 382 *w*, 208 *s* cm^−1^.

M.p.: 362 K.

## Refinement   

Crystal data, data collection and structure refinement details are summarized in Table 5 ▸. All hydrogen atoms were positioned geometrically and refined using a riding model with *U*_iso_(H) = 1.2(CH and CH_2_) or 1.5(CH_3_) times *U*_eq_(C). Reflections with error/e.s.d. > 8 were omitted. Error/e.s.d. = (*wD* ^2^/<*wD* ^2^>)^0.5^ where *D* = *F*_o_^2^ - *F*_c_^2^.

## Supplementary Material

Crystal structure: contains datablock(s) 1, 2, 3. DOI: 10.1107/S2056989019016372/wm5524sup1.cif

Structure factors: contains datablock(s) 1. DOI: 10.1107/S2056989019016372/wm55241sup2.hkl

Structure factors: contains datablock(s) 2. DOI: 10.1107/S2056989019016372/wm55242sup3.hkl

Structure factors: contains datablock(s) 3. DOI: 10.1107/S2056989019016372/wm55243sup4.hkl

CCDC references: 1969941, 1969940, 1969939

Additional supporting information:  crystallographic information; 3D view; checkCIF report

## Figures and Tables

**Figure 2:**
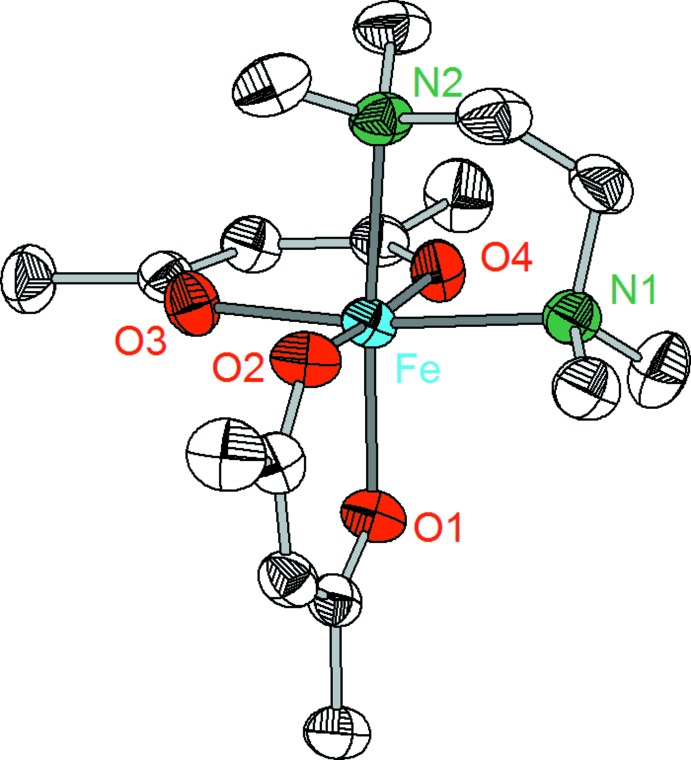
Mol­ecular structure of complex **2** showing the labeling scheme. Displacement ellipsoids drawn at 50% probability level, H atoms are omitted.

**Figure 3:**
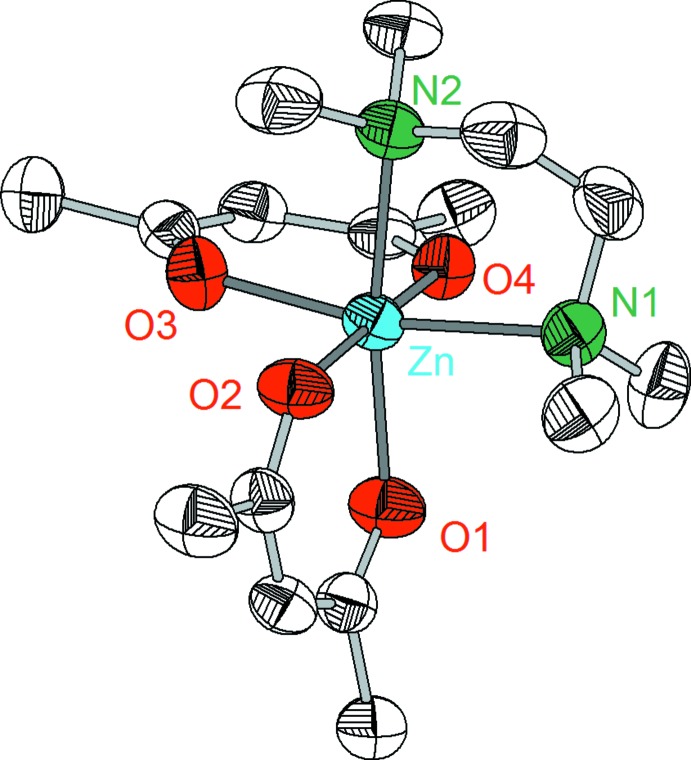
Mol­ecular structure of complex **3** showing the labeling scheme. Displacement ellipsoids drawn at 50% probability level, H atoms are omitted.

**Figure 6:**
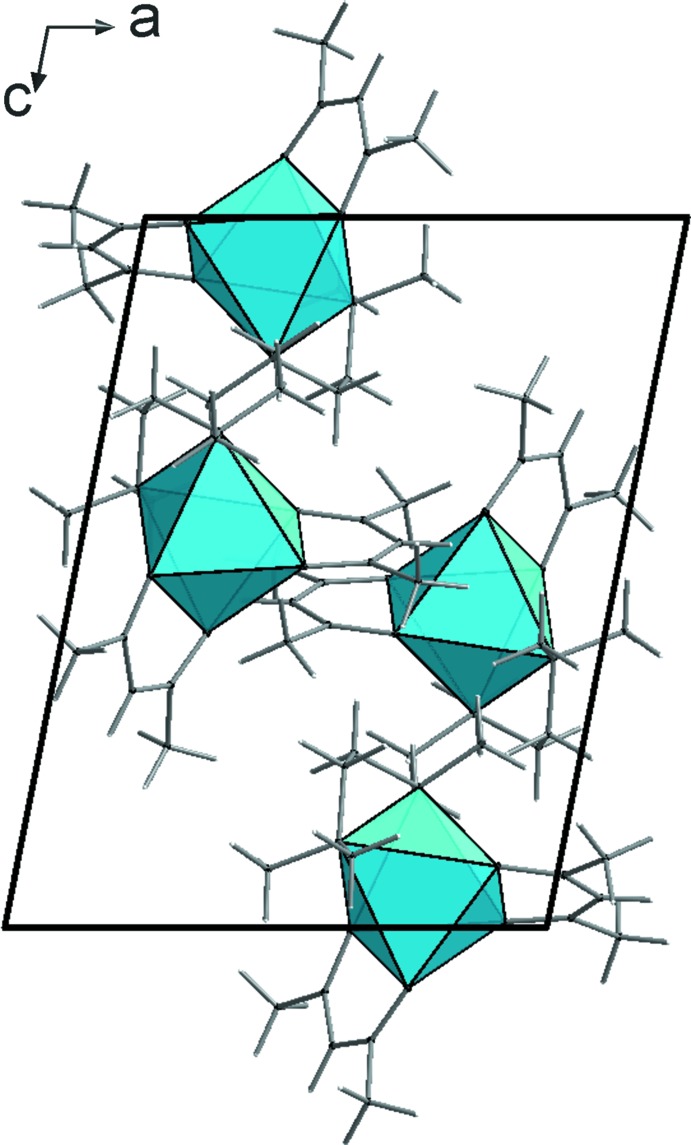
Crystal structure of compound **3**, viewed along the *b* axis.

**Table 1:** Selected geometric parameters (Å, °) for **1** [Chem scheme1]

Mn—O1	2.1271 (13)	Mn—O4	2.1365 (12)
Mn—O2	2.1500 (12)	Mn—N1	2.3643 (15)
Mn—O3	2.1375 (12)	Mn—N2	2.3560 (15)
			
O1—Mn—O2	83.61 (5)	O2—Mn—N2	90.36 (5)
O1—Mn—O3	107.00 (5)	O3—Mn—O4	83.78 (5)
O1—Mn—O4	93.25 (5)	O3—Mn—N1	165.43 (5)
O1—Mn—N1	86.01 (5)	O3—Mn—N2	90.61 (5)
O1—Mn—N2	161.29 (6)	O4—Mn—N1	89.07 (5)
O2—Mn—O3	89.71 (5)	O4—Mn—N2	94.95 (6)
O2—Mn—O4	171.63 (5)	N1—Mn—N2	77.34 (6)
O2—Mn—N1	98.41 (5)		

**Table 2:** Selected geometric parameters (Å, °) for **2** [Chem scheme1]

Fe—O1	2.0876 (10)	Fe—O4	2.0520 (9)
Fe—O2	2.0497 (10)	Fe—N1	2.3021 (12)
Fe—O3	2.0970 (10)	Fe—N2	2.3184 (12)
			
O1—Fe—O2	85.58 (4)	O2—Fe—N2	84.18 (4)
O1—Fe—O3	93.98 (4)	O3—Fe—O4	86.00 (4)
O1—Fe—O4	99.11 (4)	O3—Fe—N1	170.93 (4)
O1—Fe—N1	92.44 (4)	O3—Fe—N2	95.43 (4)
O1—Fe—N2	166.73 (4)	O4—Fe—N1	86.66 (4)
O2—Fe—O3	95.84 (4)	O4—Fe—N2	90.87 (4)
O2—Fe—O4	174.85 (4)	N1—Fe—N2	79.35 (4)
O2—Fe—N1	91.04 (5)		

**Table 3:** Selected geometric parameters (Å, °) for **3** [Chem scheme1]

Zn—O1	2.0771 (12)	Zn—O4	2.0607 (10)
Zn—O2	2.0611 (11)	Zn—N1	2.2722 (13)
Zn—O3	2.0645 (11)	Zn—N2	2.2533 (13)
			
O1—Zn—O2	87.50 (4)	O2—Zn—N2	89.57 (5)
O1—Zn—O3	101.58 (5)	O3—Zn—O4	87.96 (4)
O1—Zn—O4	88.49 (4)	O3—Zn—N1	168.61 (5)
O1—Zn—N1	89.28 (5)	O3—Zn—N2	89.09 (5)
O1—Zn—N2	168.94 (5)	O4—Zn—N1	88.92 (5)
O2—Zn—O3	90.18 (5)	O4—Zn—N2	94.86 (5)
O2—Zn—O4	175.16 (4)	N1—Zn—N2	80.27 (5)
O2—Zn—N1	93.76 (5)		

**Table 4:** Hydrogen-bond geometry (Å, °) for **2** [Chem scheme1]

*D*—H⋯*A*	*D*—H	H⋯*A*	*D*⋯*A*	*D*—H⋯*A*
C1—H2⋯O1^i^	0.96	2.62	3.5269 (18)	157

Symmetry code: (i) 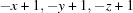.

**Table 5:** Experimental details

	**1**	**2**	**3**
Crystal data			
Chemical formula	[Mn(C_5_H_7_O_2_)_2_(C_6_H_16_N_2_)]	[Fe(C_5_H_7_O_2_)_2_(C_6_H_16_N_2_)]	[Zn(C_5_H_7_O_2_)_2_(C_6_H_16_N_2_)]
*M*_r_	369.36	370.27	379.79
Crystal system, space group	Monoclinic, *P*2_1_/*n*	Monoclinic, *P*2_1_/*n*	Monoclinic, *P*2_1_/*n*
Temperature (K)	213	213	200
*a*, *b*, *c* (Å)	10.4234 (4), 14.3123 (5), 13.6047 (5)	10.2021 (3), 15.4708 (4), 12.4881 (4)	10.2335 (3), 14.2134 (6), 13.6738 (5)
β (°)	103.154 (3)	95.382 (3)	101.208 (3)
*V* (Å^3^)	1976.33 (13)	1962.37 (10)	1950.96 (12)
*Z*	4	4	4
Radiation type	Mo *K*α	Mo *K*α	Mo *K*α
μ (mm^−1^)	0.69	0.79	1.28
Crystal size (mm)	0.35 × 0.25 × 0.20	0.26 × 0.25 × 0.23	0.45 × 0.39 × 0.33

Data collection			
Diffractometer	STOE IPDS 2	STOE IPDS 2	STOE IPDS 2T
Absorption correction	Numerical (*X-AREA*; Stoe & Cie, 2016 ▸)	Numerical (*X-AREA*; Stoe & Cie, 2016 ▸)	Numerical (*X-AREA*; Stoe & Cie, 2016 ▸)
*T*_min_, *T*_max_	0.798, 0.912	0.814, 0.894	0.627, 0.779
No. of measured, independent and observed [*I* > 2σ(*I*)] reflections	12607, 4139, 3475	18586, 5276, 4425	22385, 4124, 3456
*R*_int_	0.030	0.037	0.047
(sin θ/λ)_max_ (Å^−1^)	0.634	0.688	0.633

Refinement			
*R*[*F* ^2^ > 2σ(*F* ^2^)], *wR*(*F* ^2^), *S*	0.034, 0.099, 1.06	0.031, 0.086, 1.04	0.027, 0.076, 1.07
No. of reflections	4139	5276	4124
No. of parameters	216	216	216
H-atom treatment	H-atom parameters constrained	H-atom parameters constrained	H-atom parameters constrained
Δρ_max_, Δρ_min_ (e Å^−3^)	0.22, −0.24	0.32, −0.22	0.37, −0.26

Computer programs: *X-AREA* (Stoe & Cie, 2016 ▸), *SHELXT2014/7* (Sheldrick, 2015*a* ▸), *SHELXL2014/7* (Sheldrick, 2015*b* ▸), *DIAMOND* (Brandenburg, 2019 ▸) and *OLEX2* (Dolomanov *et al.*, 2009 ▸).

## supplementary crystallographic information

### Bis(acetylacetonato-κ2O,O')(N,N,N',N'-tetramethylethylenediamine-κ2N,N')manganese(II) (1) Crystal data

**Table:**

[Mn(C_5_H_7_O_2_)_2_(C_6_H_16_N_2_)]	*F*(000) = 788
*M**_r_* = 369.36	*D*_x_ = 1.241 Mg m^−^^3^
Monoclinic, *P*2_1_/*n*	Mo *K*α radiation, λ = 0.71073 Å
*a* = 10.4234 (4) Å	Cell parameters from 13227 reflections
*b* = 14.3123 (5) Å	θ = 1.4–27.2°
*c* = 13.6047 (5) Å	µ = 0.69 mm^−^^1^
β = 103.154 (3)°	*T* = 213 K
*V* = 1976.33 (13) Å^3^	Block, clear yellow
*Z* = 4	0.35 × 0.25 × 0.20 mm

### Bis(acetylacetonato-κ2O,O')(N,N,N',N'-tetramethylethylenediamine-κ2N,N')manganese(II) (1) Data collection

**Table:**

STOE IPDS 2 diffractometer	4139 independent reflections
Radiation source: sealed X-ray tube, 12 x 0.4 mm long-fine focus, Incoatec Iµs	3475 reflections with *I* > 2σ(*I*)
Plane graphite monochromator	*R*_int_ = 0.030
Detector resolution: 6.67 pixels mm^-1^	θ_max_ = 26.8°, θ_min_ = 2.1°
rotation method scans	*h* = −13→13
Absorption correction: numerical (X-AREA; Stoe & Cie, 2016)	*k* = −17→18
*T*_min_ = 0.798, *T*_max_ = 0.912	*l* = −17→16
12607 measured reflections	

### Bis(acetylacetonato-κ2O,O')(N,N,N',N'-tetramethylethylenediamine-κ2N,N')manganese(II) (1) Refinement

**Table:**

Refinement on *F*^2^	0 restraints
Least-squares matrix: full	Hydrogen site location: inferred from neighbouring sites
*R*[*F*^2^ > 2σ(*F*^2^)] = 0.034	H-atom parameters constrained
*wR*(*F*^2^) = 0.099	*w* = 1/[σ^2^(*F*_o_^2^) + (0.0447*P*)^2^ + 0.6571*P*] where *P* = (*F*_o_^2^ + 2*F*_c_^2^)/3
*S* = 1.06	(Δ/σ)_max_ = 0.001
4139 reflections	Δρ_max_ = 0.22 e Å^−^^3^
216 parameters	Δρ_min_ = −0.24 e Å^−^^3^

### Bis(acetylacetonato-κ2O,O')(N,N,N',N'-tetramethylethylenediamine-κ2N,N')manganese(II) (1) Special details

**Table:**

Geometry. All esds (except the esd in the dihedral angle between two l.s. planes) are estimated using the full covariance matrix. The cell esds are taken into account individually in the estimation of esds in distances, angles and torsion angles; correlations between esds in cell parameters are only used when they are defined by crystal symmetry. An approximate (isotropic) treatment of cell esds is used for estimating esds involving l.s. planes.

### Bis(acetylacetonato-κ2O,O')(N,N,N',N'-tetramethylethylenediamine-κ2N,N')manganese(II) (1) Fractional atomic coordinates and isotropic or equivalent isotropic displacement parameters (Å^2^)

**Table:**

	*x*	*y*	*z*	*U*_iso_*/*U*_eq_	
Mn	0.50579 (2)	0.74721 (2)	0.49407 (2)	0.04120 (11)	
O1	0.67069 (13)	0.65614 (9)	0.53426 (10)	0.0602 (3)	
O2	0.64605 (12)	0.84003 (9)	0.45153 (11)	0.0554 (3)	
O3	0.50538 (13)	0.83274 (9)	0.62280 (10)	0.0546 (3)	
O4	0.38216 (14)	0.65820 (9)	0.55845 (10)	0.0565 (3)	
N1	0.44810 (15)	0.65991 (11)	0.34289 (11)	0.0523 (4)	
N2	0.33093 (14)	0.83481 (11)	0.39666 (12)	0.0533 (4)	
C1	0.8801 (3)	0.5868 (2)	0.5617 (2)	0.0943 (9)	
H1	0.8477	0.5484	0.6086	0.141*	
H3	0.9667	0.6089	0.5928	0.141*	
H2	0.8838	0.5506	0.5029	0.141*	
C2	0.7888 (2)	0.66910 (16)	0.53114 (14)	0.0593 (5)	
C3	0.8405 (2)	0.75159 (16)	0.50286 (17)	0.0664 (6)	
H4	0.9308	0.7532	0.5070	0.080*	
C4	0.76938 (18)	0.83223 (14)	0.46880 (15)	0.0568 (5)	
C5	0.8448 (2)	0.91826 (18)	0.4505 (2)	0.0885 (8)	
H5	0.9136	0.9006	0.4178	0.133*	
H6	0.8827	0.9477	0.5138	0.133*	
H7	0.7860	0.9611	0.4082	0.133*	
C6	0.4607 (2)	0.89848 (16)	0.77048 (18)	0.0714 (6)	
H8	0.5521	0.9144	0.7936	0.107*	
H10	0.4263	0.8782	0.8267	0.107*	
H9	0.4125	0.9522	0.7400	0.107*	
C7	0.44674 (17)	0.82045 (13)	0.69347 (13)	0.0498 (4)	
C8	0.3712 (2)	0.74328 (13)	0.70557 (16)	0.0568 (5)	
H11	0.3367	0.7416	0.7629	0.068*	
C9	0.34284 (19)	0.66822 (13)	0.63927 (15)	0.0558 (4)	
C10	0.2576 (3)	0.59062 (18)	0.6645 (2)	0.0920 (9)	
H12	0.2171	0.6107	0.7176	0.138*	
H13	0.3109	0.5365	0.6861	0.138*	
H14	0.1904	0.5754	0.6057	0.138*	
C11	0.3189 (2)	0.69516 (18)	0.28808 (16)	0.0693 (6)	
H16	0.2506	0.6684	0.3173	0.083*	
H15	0.3030	0.6753	0.2182	0.083*	
C12	0.3114 (2)	0.79961 (18)	0.29216 (16)	0.0703 (6)	
H17	0.3781	0.8263	0.2612	0.084*	
H18	0.2260	0.8199	0.2535	0.084*	
C13	0.4397 (3)	0.55956 (14)	0.36410 (18)	0.0728 (6)	
H20	0.3743	0.5496	0.4026	0.109*	
H21	0.5236	0.5378	0.4018	0.109*	
H19	0.4156	0.5258	0.3016	0.109*	
C14	0.5481 (2)	0.67378 (17)	0.28339 (15)	0.0647 (5)	
H24	0.5227	0.6403	0.2208	0.097*	
H22	0.6316	0.6509	0.3207	0.097*	
H23	0.5553	0.7392	0.2698	0.097*	
C15	0.21051 (19)	0.82208 (18)	0.43395 (18)	0.0711 (6)	
H25	0.1889	0.7568	0.4331	0.107*	
H27	0.1393	0.8557	0.3914	0.107*	
H26	0.2246	0.8454	0.5017	0.107*	
C16	0.3638 (2)	0.93462 (15)	0.4003 (2)	0.0784 (7)	
H29	0.2945	0.9685	0.3560	0.118*	
H28	0.4448	0.9435	0.3793	0.118*	
H30	0.3736	0.9572	0.4680	0.118*	

### Bis(acetylacetonato-κ2O,O')(N,N,N',N'-tetramethylethylenediamine-κ2N,N')manganese(II) (1) Atomic displacement parameters (Å^2^)

**Table:**

	*U*^11^	*U*^22^	*U*^33^	*U*^12^	*U*^13^	*U*^23^
Mn	0.03895 (15)	0.04541 (18)	0.03899 (15)	−0.00103 (10)	0.00838 (10)	0.00066 (10)
O1	0.0612 (8)	0.0613 (8)	0.0561 (8)	0.0159 (6)	0.0090 (6)	0.0076 (6)
O2	0.0433 (6)	0.0523 (7)	0.0725 (9)	−0.0029 (5)	0.0168 (6)	0.0043 (6)
O3	0.0591 (7)	0.0537 (7)	0.0526 (7)	−0.0101 (6)	0.0160 (6)	−0.0103 (6)
O4	0.0700 (8)	0.0495 (7)	0.0567 (8)	−0.0136 (6)	0.0282 (6)	−0.0085 (6)
N1	0.0538 (8)	0.0612 (9)	0.0426 (8)	−0.0076 (7)	0.0124 (6)	−0.0048 (7)
N2	0.0431 (7)	0.0595 (9)	0.0546 (9)	0.0040 (7)	0.0054 (6)	0.0063 (7)
C1	0.0924 (18)	0.108 (2)	0.0744 (15)	0.0575 (16)	0.0027 (13)	−0.0049 (14)
C2	0.0561 (11)	0.0758 (14)	0.0411 (9)	0.0216 (10)	0.0009 (8)	−0.0109 (9)
C3	0.0392 (9)	0.0933 (17)	0.0658 (13)	0.0089 (10)	0.0100 (9)	−0.0196 (11)
C4	0.0454 (9)	0.0699 (12)	0.0589 (11)	−0.0094 (9)	0.0197 (8)	−0.0190 (9)
C5	0.0657 (14)	0.0870 (17)	0.124 (2)	−0.0267 (13)	0.0450 (15)	−0.0239 (16)
C6	0.0770 (14)	0.0710 (14)	0.0676 (13)	0.0026 (11)	0.0191 (11)	−0.0237 (11)
C7	0.0473 (9)	0.0556 (10)	0.0450 (9)	0.0070 (8)	0.0076 (7)	−0.0062 (8)
C8	0.0628 (12)	0.0614 (12)	0.0525 (10)	−0.0014 (9)	0.0262 (9)	−0.0058 (8)
C9	0.0581 (10)	0.0560 (11)	0.0590 (11)	−0.0039 (9)	0.0253 (9)	−0.0014 (9)
C10	0.110 (2)	0.0822 (17)	0.104 (2)	−0.0364 (15)	0.0658 (17)	−0.0179 (15)
C11	0.0544 (11)	0.0974 (17)	0.0505 (11)	−0.0062 (11)	0.0004 (9)	−0.0157 (11)
C12	0.0594 (12)	0.0975 (17)	0.0483 (11)	0.0137 (11)	0.0000 (9)	0.0114 (11)
C13	0.1012 (17)	0.0554 (12)	0.0660 (13)	−0.0177 (11)	0.0276 (12)	−0.0174 (10)
C14	0.0666 (12)	0.0849 (15)	0.0466 (10)	−0.0047 (11)	0.0210 (9)	−0.0051 (10)
C15	0.0432 (10)	0.0931 (16)	0.0760 (14)	0.0085 (10)	0.0118 (9)	0.0033 (12)
C16	0.0653 (13)	0.0614 (13)	0.1024 (19)	0.0125 (10)	0.0063 (12)	0.0203 (12)

### Bis(acetylacetonato-κ2O,O')(N,N,N',N'-tetramethylethylenediamine-κ2N,N')manganese(II) (1) Geometric parameters (Å, º)

**Table:**

Mn—O1	2.1271 (13)	C6—H10	0.9600
Mn—O2	2.1500 (12)	C6—H9	0.9600
Mn—O3	2.1375 (12)	C6—C7	1.515 (3)
Mn—O4	2.1365 (12)	C7—C8	1.388 (3)
Mn—N1	2.3643 (15)	C8—H11	0.9300
Mn—N2	2.3560 (15)	C8—C9	1.391 (3)
O1—C2	1.255 (2)	C9—C10	1.510 (3)
O2—C4	1.258 (2)	C10—H12	0.9600
O3—C7	1.263 (2)	C10—H13	0.9600
O4—C9	1.266 (2)	C10—H14	0.9600
N1—C11	1.472 (3)	C11—H16	0.9700
N1—C13	1.472 (3)	C11—H15	0.9700
N1—C14	1.472 (2)	C11—C12	1.499 (4)
N2—C12	1.478 (3)	C12—H17	0.9700
N2—C15	1.468 (2)	C12—H18	0.9700
N2—C16	1.467 (3)	C13—H20	0.9600
C1—H1	0.9600	C13—H21	0.9600
C1—H3	0.9600	C13—H19	0.9600
C1—H2	0.9600	C14—H24	0.9600
C1—C2	1.512 (3)	C14—H22	0.9600
C2—C3	1.388 (3)	C14—H23	0.9600
C3—H4	0.9300	C15—H25	0.9600
C3—C4	1.393 (3)	C15—H27	0.9600
C4—C5	1.512 (3)	C15—H26	0.9600
C5—H5	0.9600	C16—H29	0.9600
C5—H6	0.9600	C16—H28	0.9600
C5—H7	0.9600	C16—H30	0.9600
C6—H8	0.9600		
			
O1—Mn—O2	83.61 (5)	C7—C6—H8	109.5
O1—Mn—O3	107.00 (5)	C7—C6—H10	109.5
O1—Mn—O4	93.25 (5)	C7—C6—H9	109.5
O1—Mn—N1	86.01 (5)	O3—C7—C6	115.89 (17)
O1—Mn—N2	161.29 (6)	O3—C7—C8	125.92 (17)
O2—Mn—O3	89.71 (5)	C8—C7—C6	118.18 (17)
O2—Mn—O4	171.63 (5)	C7—C8—H11	117.2
O2—Mn—N1	98.41 (5)	C7—C8—C9	125.50 (18)
O2—Mn—N2	90.36 (5)	C9—C8—H11	117.2
O3—Mn—O4	83.78 (5)	O4—C9—C8	125.99 (17)
O3—Mn—N1	165.43 (5)	O4—C9—C10	115.94 (18)
O3—Mn—N2	90.61 (5)	C8—C9—C10	118.07 (18)
O4—Mn—N1	89.07 (5)	C9—C10—H12	109.5
O4—Mn—N2	94.95 (6)	C9—C10—H13	109.5
N1—Mn—N2	77.34 (6)	C9—C10—H14	109.5
C2—O1—Mn	129.95 (14)	H12—C10—H13	109.5
C4—O2—Mn	128.56 (13)	H12—C10—H14	109.5
C7—O3—Mn	129.44 (12)	H13—C10—H14	109.5
C9—O4—Mn	129.29 (12)	N1—C11—H16	109.2
C11—N1—Mn	106.37 (12)	N1—C11—H15	109.2
C13—N1—Mn	111.10 (12)	N1—C11—C12	111.88 (17)
C13—N1—C11	110.20 (18)	H16—C11—H15	107.9
C13—N1—C14	108.71 (17)	C12—C11—H16	109.2
C14—N1—Mn	109.60 (12)	C12—C11—H15	109.2
C14—N1—C11	110.86 (16)	N2—C12—C11	112.25 (17)
C12—N2—Mn	106.22 (12)	N2—C12—H17	109.2
C15—N2—Mn	110.63 (12)	N2—C12—H18	109.2
C15—N2—C12	110.40 (17)	C11—C12—H17	109.2
C16—N2—Mn	110.75 (12)	C11—C12—H18	109.2
C16—N2—C12	110.14 (18)	H17—C12—H18	107.9
C16—N2—C15	108.69 (17)	N1—C13—H20	109.5
H1—C1—H3	109.5	N1—C13—H21	109.5
H1—C1—H2	109.5	N1—C13—H19	109.5
H3—C1—H2	109.5	H20—C13—H21	109.5
C2—C1—H1	109.5	H20—C13—H19	109.5
C2—C1—H3	109.5	H21—C13—H19	109.5
C2—C1—H2	109.5	N1—C14—H24	109.5
O1—C2—C1	115.9 (2)	N1—C14—H22	109.5
O1—C2—C3	125.51 (18)	N1—C14—H23	109.5
C3—C2—C1	118.6 (2)	H24—C14—H22	109.5
C2—C3—H4	117.1	H24—C14—H23	109.5
C2—C3—C4	125.86 (18)	H22—C14—H23	109.5
C4—C3—H4	117.1	N2—C15—H25	109.5
O2—C4—C3	125.44 (19)	N2—C15—H27	109.5
O2—C4—C5	116.4 (2)	N2—C15—H26	109.5
C3—C4—C5	118.17 (19)	H25—C15—H27	109.5
C4—C5—H5	109.5	H25—C15—H26	109.5
C4—C5—H6	109.5	H27—C15—H26	109.5
C4—C5—H7	109.5	N2—C16—H29	109.5
H5—C5—H6	109.5	N2—C16—H28	109.5
H5—C5—H7	109.5	N2—C16—H30	109.5
H6—C5—H7	109.5	H29—C16—H28	109.5
H8—C6—H10	109.5	H29—C16—H30	109.5
H8—C6—H9	109.5	H28—C16—H30	109.5
H10—C6—H9	109.5		
			
Mn—O1—C2—C1	−177.50 (14)	N1—C11—C12—N2	−60.6 (2)
Mn—O1—C2—C3	2.8 (3)	C1—C2—C3—C4	177.1 (2)
Mn—O2—C4—C3	13.4 (3)	C2—C3—C4—O2	−5.6 (3)
Mn—O2—C4—C5	−166.67 (16)	C2—C3—C4—C5	174.5 (2)
Mn—O3—C7—C6	−176.26 (13)	C6—C7—C8—C9	176.5 (2)
Mn—O3—C7—C8	3.2 (3)	C7—C8—C9—O4	0.7 (4)
Mn—O4—C9—C8	1.1 (3)	C7—C8—C9—C10	−179.4 (2)
Mn—O4—C9—C10	−178.82 (17)	C13—N1—C11—C12	162.74 (17)
Mn—N1—C11—C12	42.22 (19)	C14—N1—C11—C12	−76.9 (2)
Mn—N2—C12—C11	42.41 (19)	C15—N2—C12—C11	−77.6 (2)
O1—C2—C3—C4	−3.2 (3)	C16—N2—C12—C11	162.39 (17)
O3—C7—C8—C9	−3.0 (3)		

### Bis(acetylacetonato-κ2O,O')(N,N,N',N'-tetramethylethylenediamine-κ2N,N')iron(II) (2) Crystal data

**Table:**

[Fe(C_5_H_7_O_2_)_2_(C_6_H_16_N_2_)]	*F*(000) = 792
*M**_r_* = 370.27	*D*_x_ = 1.253 Mg m^−^^3^
Monoclinic, *P*2_1_/*n*	Mo *K*α radiation, λ = 0.71073 Å
*a* = 10.2021 (3) Å	Cell parameters from 16780 reflections
*b* = 15.4708 (4) Å	θ = 1.6–29.6°
*c* = 12.4881 (4) Å	µ = 0.79 mm^−^^1^
β = 95.382 (3)°	*T* = 213 K
*V* = 1962.37 (10) Å^3^	Block, clear reddish brown
*Z* = 4	0.26 × 0.25 × 0.23 mm

### Bis(acetylacetonato-κ2O,O')(N,N,N',N'-tetramethylethylenediamine-κ2N,N')iron(II) (2) Data collection

**Table:**

STOE IPDS 2 diffractometer	5276 independent reflections
Radiation source: sealed X-ray tube, 12 x 0.4 mm long-fine focus, Incoatec Iµs	4425 reflections with *I* > 2σ(*I*)
Plane graphite monochromator	*R*_int_ = 0.037
Detector resolution: 6.67 pixels mm^-1^	θ_max_ = 29.3°, θ_min_ = 2.1°
rotation method scans	*h* = −13→13
Absorption correction: numerical (X-AREA; Stoe & Cie, 2016)	*k* = −21→20
*T*_min_ = 0.814, *T*_max_ = 0.894	*l* = −17→17
18586 measured reflections	

### Bis(acetylacetonato-κ2O,O')(N,N,N',N'-tetramethylethylenediamine-κ2N,N')iron(II) (2) Refinement

**Table:**

Refinement on *F*^2^	0 restraints
Least-squares matrix: full	Hydrogen site location: inferred from neighbouring sites
*R*[*F*^2^ > 2σ(*F*^2^)] = 0.031	H-atom parameters constrained
*wR*(*F*^2^) = 0.086	*w* = 1/[σ^2^(*F*_o_^2^) + (0.0464*P*)^2^ + 0.3681*P*] where *P* = (*F*_o_^2^ + 2*F*_c_^2^)/3
*S* = 1.04	(Δ/σ)_max_ = 0.002
5276 reflections	Δρ_max_ = 0.32 e Å^−^^3^
216 parameters	Δρ_min_ = −0.22 e Å^−^^3^

### Bis(acetylacetonato-κ2O,O')(N,N,N',N'-tetramethylethylenediamine-κ2N,N')iron(II) (2) Special details

**Table:**

Geometry. All esds (except the esd in the dihedral angle between two l.s. planes) are estimated using the full covariance matrix. The cell esds are taken into account individually in the estimation of esds in distances, angles and torsion angles; correlations between esds in cell parameters are only used when they are defined by crystal symmetry. An approximate (isotropic) treatment of cell esds is used for estimating esds involving l.s. planes.

### Bis(acetylacetonato-κ2O,O')(N,N,N',N'-tetramethylethylenediamine-κ2N,N')iron(II) (2) Fractional atomic coordinates and isotropic or equivalent isotropic displacement parameters (Å^2^)

**Table:**

	*x*	*y*	*z*	*U*_iso_*/*U*_eq_	
C1	0.63613 (15)	0.53037 (11)	0.42322 (12)	0.0444 (3)	
H2	0.5847	0.4797	0.4338	0.067*	
H3	0.7061	0.5160	0.3800	0.067*	
H1	0.5810	0.5739	0.3874	0.067*	
C2	0.69359 (13)	0.56425 (9)	0.53071 (11)	0.0352 (3)	
C3	0.81769 (14)	0.53357 (9)	0.57243 (12)	0.0396 (3)	
H4	0.8603	0.4948	0.5305	0.047*	
C4	0.88149 (13)	0.55660 (9)	0.67116 (12)	0.0388 (3)	
C5	1.01582 (16)	0.51923 (13)	0.70525 (16)	0.0584 (4)	
H7	1.0343	0.4733	0.6572	0.088*	
H5	1.0172	0.4970	0.7771	0.088*	
H6	1.0813	0.5636	0.7031	0.088*	
C6	0.39240 (17)	0.48770 (10)	0.87153 (14)	0.0496 (4)	
H10	0.3000	0.4817	0.8499	0.074*	
H9	0.4064	0.4888	0.9486	0.074*	
H8	0.4391	0.4397	0.8446	0.074*	
C7	0.44227 (13)	0.57093 (9)	0.82668 (11)	0.0359 (3)	
C8	0.35444 (13)	0.64029 (10)	0.81410 (12)	0.0379 (3)	
H11	0.2707	0.6321	0.8359	0.046*	
C9	0.38200 (13)	0.72026 (9)	0.77178 (11)	0.0350 (3)	
C10	0.27918 (16)	0.79023 (12)	0.77050 (16)	0.0542 (4)	
H14	0.2694	0.8171	0.7009	0.081*	
H13	0.3059	0.8328	0.8241	0.081*	
H12	0.1967	0.7654	0.7857	0.081*	
C11	0.78186 (19)	0.85071 (11)	0.76164 (14)	0.0537 (4)	
H16	0.7020	0.8793	0.7792	0.064*	
H15	0.8440	0.8949	0.7446	0.064*	
C12	0.83901 (17)	0.80004 (13)	0.85721 (14)	0.0545 (4)	
H17	0.9192	0.7718	0.8398	0.065*	
H18	0.8617	0.8393	0.9167	0.065*	
C13	0.6596 (2)	0.83936 (13)	0.58754 (16)	0.0593 (4)	
H19	0.6381	0.8022	0.5269	0.089*	
H21	0.7000	0.8912	0.5641	0.089*	
H20	0.5807	0.8539	0.6198	0.089*	
C14	0.87086 (16)	0.77473 (12)	0.61406 (14)	0.0519 (4)	
H24	0.9042	0.8268	0.5847	0.078*	
H22	0.8499	0.7338	0.5572	0.078*	
H23	0.9363	0.7506	0.6658	0.078*	
C15	0.64471 (18)	0.77489 (13)	0.94987 (14)	0.0549 (4)	
H26	0.5825	0.7319	0.9677	0.082*	
H25	0.6001	0.8188	0.9060	0.082*	
H27	0.6853	0.8005	1.0147	0.082*	
C16	0.8174 (2)	0.67090 (14)	0.96194 (14)	0.0619 (5)	
H29	0.8834	0.6428	0.9244	0.093*	
H28	0.7566	0.6286	0.9840	0.093*	
H30	0.8587	0.7000	1.0242	0.093*	
Fe	0.65967 (2)	0.67126 (2)	0.73081 (2)	0.03242 (7)	
N1	0.75139 (12)	0.79468 (8)	0.66685 (10)	0.0407 (3)	
N2	0.74634 (12)	0.73421 (9)	0.89046 (10)	0.0418 (3)	
O1	0.62568 (9)	0.61851 (7)	0.57684 (8)	0.0395 (2)	
O2	0.83743 (9)	0.60880 (7)	0.73746 (9)	0.0426 (2)	
O3	0.55981 (10)	0.57212 (6)	0.80388 (9)	0.0414 (2)	
O4	0.48936 (9)	0.74134 (6)	0.73429 (8)	0.0384 (2)	

### Bis(acetylacetonato-κ2O,O')(N,N,N',N'-tetramethylethylenediamine-κ2N,N')iron(II) (2) Atomic displacement parameters (Å^2^)

**Table:**

	*U*^11^	*U*^22^	*U*^33^	*U*^12^	*U*^13^	*U*^23^
C1	0.0435 (7)	0.0512 (8)	0.0393 (7)	−0.0086 (6)	0.0082 (6)	−0.0108 (6)
C2	0.0360 (6)	0.0341 (6)	0.0368 (7)	−0.0081 (5)	0.0096 (5)	−0.0039 (5)
C3	0.0380 (7)	0.0368 (7)	0.0453 (8)	0.0022 (5)	0.0111 (6)	−0.0064 (6)
C4	0.0301 (6)	0.0407 (7)	0.0465 (8)	0.0000 (5)	0.0089 (5)	0.0000 (6)
C5	0.0374 (8)	0.0703 (12)	0.0670 (11)	0.0127 (8)	0.0019 (7)	−0.0056 (9)
C6	0.0539 (9)	0.0434 (8)	0.0515 (9)	−0.0121 (7)	0.0050 (7)	0.0076 (7)
C7	0.0380 (6)	0.0369 (7)	0.0328 (6)	−0.0079 (5)	0.0028 (5)	−0.0029 (5)
C8	0.0303 (6)	0.0435 (7)	0.0413 (7)	−0.0049 (5)	0.0102 (5)	−0.0045 (6)
C9	0.0315 (6)	0.0393 (7)	0.0349 (6)	0.0017 (5)	0.0056 (5)	−0.0047 (5)
C10	0.0439 (8)	0.0539 (9)	0.0670 (11)	0.0149 (7)	0.0163 (7)	0.0022 (8)
C11	0.0674 (11)	0.0428 (8)	0.0540 (10)	−0.0207 (8)	0.0222 (8)	−0.0125 (7)
C12	0.0508 (9)	0.0685 (11)	0.0451 (9)	−0.0275 (8)	0.0092 (7)	−0.0158 (8)
C13	0.0626 (11)	0.0614 (11)	0.0559 (10)	−0.0011 (8)	0.0154 (8)	0.0167 (8)
C14	0.0496 (8)	0.0561 (9)	0.0537 (9)	−0.0149 (7)	0.0247 (7)	−0.0073 (7)
C15	0.0550 (9)	0.0690 (11)	0.0429 (8)	−0.0094 (8)	0.0167 (7)	−0.0164 (8)
C16	0.0650 (11)	0.0792 (13)	0.0401 (9)	0.0011 (9)	−0.0027 (8)	−0.0002 (8)
Fe	0.02688 (10)	0.03490 (11)	0.03626 (11)	−0.00268 (7)	0.00714 (7)	−0.00567 (7)
N1	0.0432 (6)	0.0415 (6)	0.0395 (6)	−0.0086 (5)	0.0147 (5)	−0.0039 (5)
N2	0.0395 (6)	0.0513 (7)	0.0354 (6)	−0.0101 (5)	0.0077 (5)	−0.0055 (5)
O1	0.0329 (5)	0.0440 (5)	0.0416 (5)	0.0005 (4)	0.0028 (4)	−0.0112 (4)
O2	0.0313 (5)	0.0535 (6)	0.0429 (5)	0.0021 (4)	0.0031 (4)	−0.0100 (5)
O3	0.0352 (5)	0.0352 (5)	0.0543 (6)	−0.0009 (4)	0.0075 (4)	0.0014 (4)
O4	0.0349 (5)	0.0348 (5)	0.0471 (5)	0.0018 (4)	0.0124 (4)	0.0023 (4)

### Bis(acetylacetonato-κ2O,O')(N,N,N',N'-tetramethylethylenediamine-κ2N,N')iron(II) (2) Geometric parameters (Å, º)

**Table:**

C1—H2	0.9600	C11—C12	1.499 (3)
C1—H3	0.9600	C11—N1	1.477 (2)
C1—H1	0.9600	C12—H17	0.9700
C1—C2	1.5076 (19)	C12—H18	0.9700
C2—C3	1.406 (2)	C12—N2	1.475 (2)
C2—O1	1.2615 (16)	C13—H19	0.9600
C3—H4	0.9300	C13—H21	0.9600
C3—C4	1.386 (2)	C13—H20	0.9600
C4—C5	1.511 (2)	C13—N1	1.471 (2)
C4—O2	1.2687 (17)	C14—H24	0.9600
C5—H7	0.9600	C14—H22	0.9600
C5—H5	0.9600	C14—H23	0.9600
C5—H6	0.9600	C14—N1	1.4718 (19)
C6—H10	0.9600	C15—H26	0.9600
C6—H9	0.9600	C15—H25	0.9600
C6—H8	0.9600	C15—H27	0.9600
C6—C7	1.511 (2)	C15—N2	1.472 (2)
C7—C8	1.397 (2)	C16—H29	0.9600
C7—O3	1.2583 (17)	C16—H28	0.9600
C8—H11	0.9300	C16—H30	0.9600
C8—C9	1.385 (2)	C16—N2	1.470 (2)
C9—C10	1.506 (2)	Fe—O1	2.0876 (10)
C9—O4	1.2734 (16)	Fe—O2	2.0497 (10)
C10—H14	0.9600	Fe—O3	2.0970 (10)
C10—H13	0.9600	Fe—O4	2.0520 (9)
C10—H12	0.9600	Fe—N1	2.3021 (12)
C11—H16	0.9700	Fe—N2	2.3184 (12)
C11—H15	0.9700		
			
H2—C1—H3	109.5	H19—C13—H20	109.5
H2—C1—H1	109.5	H21—C13—H20	109.5
H3—C1—H1	109.5	N1—C13—H19	109.5
C2—C1—H2	109.5	N1—C13—H21	109.5
C2—C1—H3	109.5	N1—C13—H20	109.5
C2—C1—H1	109.5	H24—C14—H22	109.5
C3—C2—C1	118.30 (12)	H24—C14—H23	109.5
O1—C2—C1	116.98 (13)	H22—C14—H23	109.5
O1—C2—C3	124.72 (13)	N1—C14—H24	109.5
C2—C3—H4	117.5	N1—C14—H22	109.5
C4—C3—C2	125.07 (13)	N1—C14—H23	109.5
C4—C3—H4	117.5	H26—C15—H25	109.5
C3—C4—C5	119.35 (14)	H26—C15—H27	109.5
O2—C4—C3	125.36 (13)	H25—C15—H27	109.5
O2—C4—C5	115.28 (14)	N2—C15—H26	109.5
C4—C5—H7	109.5	N2—C15—H25	109.5
C4—C5—H5	109.5	N2—C15—H27	109.5
C4—C5—H6	109.5	H29—C16—H28	109.5
H7—C5—H5	109.5	H29—C16—H30	109.5
H7—C5—H6	109.5	H28—C16—H30	109.5
H5—C5—H6	109.5	N2—C16—H29	109.5
H10—C6—H9	109.5	N2—C16—H28	109.5
H10—C6—H8	109.5	N2—C16—H30	109.5
H9—C6—H8	109.5	O1—Fe—O2	85.58 (4)
C7—C6—H10	109.5	O1—Fe—O3	93.98 (4)
C7—C6—H9	109.5	O1—Fe—O4	99.11 (4)
C7—C6—H8	109.5	O1—Fe—N1	92.44 (4)
C8—C7—C6	117.47 (13)	O1—Fe—N2	166.73 (4)
O3—C7—C6	117.22 (13)	O2—Fe—O3	95.84 (4)
O3—C7—C8	125.31 (13)	O2—Fe—O4	174.85 (4)
C7—C8—H11	117.3	O2—Fe—N1	91.04 (5)
C9—C8—C7	125.32 (12)	O2—Fe—N2	84.18 (4)
C9—C8—H11	117.3	O3—Fe—O4	86.00 (4)
C8—C9—C10	118.69 (13)	O3—Fe—N1	170.93 (4)
O4—C9—C8	125.57 (12)	O3—Fe—N2	95.43 (4)
O4—C9—C10	115.74 (13)	O4—Fe—N1	86.66 (4)
C9—C10—H14	109.5	O4—Fe—N2	90.87 (4)
C9—C10—H13	109.5	N1—Fe—N2	79.35 (4)
C9—C10—H12	109.5	C11—N1—Fe	105.70 (9)
H14—C10—H13	109.5	C13—N1—C11	109.69 (14)
H14—C10—H12	109.5	C13—N1—C14	107.39 (13)
H13—C10—H12	109.5	C13—N1—Fe	111.69 (10)
H16—C11—H15	108.0	C14—N1—C11	111.18 (13)
C12—C11—H16	109.3	C14—N1—Fe	111.24 (10)
C12—C11—H15	109.3	C12—N2—Fe	104.52 (9)
N1—C11—H16	109.3	C15—N2—C12	110.31 (14)
N1—C11—H15	109.3	C15—N2—Fe	112.54 (10)
N1—C11—C12	111.60 (14)	C16—N2—C12	109.77 (14)
C11—C12—H17	109.2	C16—N2—C15	108.03 (14)
C11—C12—H18	109.2	C16—N2—Fe	111.65 (10)
H17—C12—H18	107.9	C2—O1—Fe	129.04 (9)
N2—C12—C11	111.91 (13)	C4—O2—Fe	129.79 (9)
N2—C12—H17	109.2	C7—O3—Fe	128.42 (10)
N2—C12—H18	109.2	C9—O4—Fe	129.18 (9)
H19—C13—H21	109.5		

### Bis(acetylacetonato-κ2O,O')(N,N,N',N'-tetramethylethylenediamine-κ2N,N')iron(II) (2) Hydrogen-bond geometry (Å, º)

**Table:**

*D*—H···*A*	*D*—H	H···*A*	*D*···*A*	*D*—H···*A*
C1—H2···O1^i^	0.96	2.62	3.5269 (18)	157

Symmetry code: (i) −*x*+1, −*y*+1, −*z*+1.

### Bis(acetylacetonato-κ2O,O')(N,N,N',N'-tetramethylethylenediamine-κ2N,N')zinc(II) (3) Crystal data

**Table:**

[Zn(C_5_H_7_O_2_)_2_(C_6_H_16_N_2_)]	*F*(000) = 808
*M**_r_* = 379.79	*D*_x_ = 1.293 Mg m^−^^3^
Monoclinic, *P*2_1_/*n*	Mo *K*α radiation, λ = 0.71073 Å
*a* = 10.2335 (3) Å	Cell parameters from 19126 reflections
*b* = 14.2134 (6) Å	θ = 2.1–27.2°
*c* = 13.6738 (5) Å	µ = 1.28 mm^−^^1^
β = 101.208 (3)°	*T* = 200 K
*V* = 1950.96 (12) Å^3^	Block, clear colourless
*Z* = 4	0.45 × 0.39 × 0.33 mm

### Bis(acetylacetonato-κ2O,O')(N,N,N',N'-tetramethylethylenediamine-κ2N,N')zinc(II) (3) Data collection

**Table:**

STOE IPDS 2T diffractometer	3456 reflections with *I* > 2σ(*I*)
Detector resolution: 6.67 pixels mm^-1^	*R*_int_ = 0.047
rotation method, ω scans	θ_max_ = 26.7°, θ_min_ = 2.1°
Absorption correction: numerical (X-AREA; Stoe & Cie, 2016)	*h* = −12→12
*T*_min_ = 0.627, *T*_max_ = 0.779	*k* = −17→16
22385 measured reflections	*l* = −17→17
4124 independent reflections	

### Bis(acetylacetonato-κ2O,O')(N,N,N',N'-tetramethylethylenediamine-κ2N,N')zinc(II) (3) Refinement

**Table:**

Refinement on *F*^2^	0 restraints
Least-squares matrix: full	Hydrogen site location: inferred from neighbouring sites
*R*[*F*^2^ > 2σ(*F*^2^)] = 0.027	H-atom parameters constrained
*wR*(*F*^2^) = 0.076	*w* = 1/[σ^2^(*F*_o_^2^) + (0.0494*P*)^2^] where *P* = (*F*_o_^2^ + 2*F*_c_^2^)/3
*S* = 1.07	(Δ/σ)_max_ = 0.001
4124 reflections	Δρ_max_ = 0.37 e Å^−^^3^
216 parameters	Δρ_min_ = −0.26 e Å^−^^3^

### Bis(acetylacetonato-κ2O,O')(N,N,N',N'-tetramethylethylenediamine-κ2N,N')zinc(II) (3) Special details

**Table:**

Geometry. All esds (except the esd in the dihedral angle between two l.s. planes) are estimated using the full covariance matrix. The cell esds are taken into account individually in the estimation of esds in distances, angles and torsion angles; correlations between esds in cell parameters are only used when they are defined by crystal symmetry. An approximate (isotropic) treatment of cell esds is used for estimating esds involving l.s. planes.

### Bis(acetylacetonato-κ2O,O')(N,N,N',N'-tetramethylethylenediamine-κ2N,N')zinc(II) (3) Fractional atomic coordinates and isotropic or equivalent isotropic displacement parameters (Å^2^)

**Table:**

	*x*	*y*	*z*	*U*_iso_*/*U*_eq_	
Zn	0.74026 (2)	0.26429 (2)	0.54676 (2)	0.03991 (8)	
O1	0.73445 (12)	0.33774 (8)	0.41466 (9)	0.0534 (3)	
O2	0.85986 (11)	0.16705 (8)	0.49492 (8)	0.0507 (3)	
O3	0.57869 (11)	0.17554 (8)	0.50853 (9)	0.0529 (3)	
O4	0.61242 (9)	0.36299 (8)	0.58630 (8)	0.0441 (2)	
N1	0.91461 (12)	0.35505 (11)	0.62041 (10)	0.0499 (3)	
N2	0.78674 (12)	0.19573 (11)	0.69813 (10)	0.0459 (3)	
C1	0.76595 (19)	0.38103 (13)	0.25509 (13)	0.0575 (4)	
H1	0.6725	0.4006	0.2373	0.086*	
H2	0.7922	0.3504	0.1977	0.086*	
H3	0.8222	0.4363	0.2743	0.086*	
C2	0.78276 (15)	0.31277 (12)	0.34146 (11)	0.0450 (4)	
C3	0.85137 (17)	0.22957 (12)	0.33344 (13)	0.0492 (4)	
H4	0.8764	0.2169	0.2714	0.059*	
C4	0.88628 (15)	0.16331 (12)	0.40893 (12)	0.0462 (4)	
C5	0.9672 (2)	0.07864 (15)	0.38836 (14)	0.0672 (5)	
H5	1.0493	0.0752	0.4389	0.101*	
H6	0.9896	0.0849	0.3222	0.101*	
H7	0.9149	0.0212	0.3907	0.101*	
C6	0.3632 (2)	0.11210 (15)	0.48710 (15)	0.0691 (5)	
H8	0.3797	0.0646	0.5401	0.104*	
H9	0.3758	0.0838	0.4242	0.104*	
H10	0.2717	0.1354	0.4796	0.104*	
C7	0.45930 (16)	0.19273 (13)	0.51387 (11)	0.0476 (4)	
C8	0.41127 (15)	0.27812 (12)	0.54200 (12)	0.0475 (4)	
H11	0.3180	0.2826	0.5391	0.057*	
C9	0.48712 (14)	0.35764 (11)	0.57395 (11)	0.0412 (3)	
C10	0.41684 (16)	0.44556 (13)	0.59729 (13)	0.0554 (4)	
H12	0.3393	0.4283	0.6257	0.083*	
H13	0.3875	0.4817	0.5359	0.083*	
H14	0.4780	0.4838	0.6454	0.083*	
C11	0.93211 (19)	0.33443 (17)	0.72727 (14)	0.0676 (5)	
H15	0.8644	0.3692	0.7556	0.081*	
H16	1.0212	0.3563	0.7615	0.081*	
C12	0.91917 (19)	0.23151 (16)	0.74583 (15)	0.0651 (5)	
H17	0.9887	0.1969	0.7194	0.078*	
H18	0.9335	0.2200	0.8185	0.078*	
C13	0.88618 (18)	0.45547 (14)	0.60157 (16)	0.0671 (5)	
H19	0.9629	0.4929	0.6341	0.101*	
H20	0.8076	0.4732	0.6285	0.101*	
H21	0.8691	0.4673	0.5296	0.101*	
C14	1.03600 (16)	0.33196 (16)	0.58256 (16)	0.0651 (5)	
H22	1.0212	0.3449	0.5108	0.098*	
H23	1.0573	0.2652	0.5944	0.098*	
H24	1.1102	0.3704	0.6172	0.098*	
C15	0.68709 (18)	0.21961 (14)	0.75840 (13)	0.0560 (4)	
H25	0.6835	0.2881	0.7660	0.084*	
H26	0.7118	0.1902	0.8243	0.084*	
H27	0.5995	0.1964	0.7251	0.084*	
C16	0.7902 (2)	0.09286 (14)	0.68935 (15)	0.0680 (5)	
H28	0.7024	0.0700	0.6562	0.102*	
H29	0.8137	0.0649	0.7560	0.102*	
H30	0.8568	0.0750	0.6500	0.102*	

### Bis(acetylacetonato-κ2O,O')(N,N,N',N'-tetramethylethylenediamine-κ2N,N')zinc(II) (3) Atomic displacement parameters (Å^2^)

**Table:**

	*U*^11^	*U*^22^	*U*^33^	*U*^12^	*U*^13^	*U*^23^
Zn	0.03590 (11)	0.04475 (13)	0.04087 (12)	0.00666 (7)	0.01186 (8)	−0.00208 (7)
O1	0.0628 (7)	0.0534 (7)	0.0470 (6)	0.0178 (6)	0.0187 (5)	0.0051 (5)
O2	0.0555 (6)	0.0545 (7)	0.0465 (6)	0.0158 (5)	0.0204 (5)	0.0024 (5)
O3	0.0488 (6)	0.0496 (7)	0.0592 (7)	−0.0007 (5)	0.0078 (5)	−0.0096 (5)
O4	0.0335 (5)	0.0470 (6)	0.0538 (6)	0.0028 (4)	0.0136 (4)	−0.0040 (5)
N1	0.0353 (6)	0.0602 (9)	0.0557 (8)	−0.0041 (6)	0.0125 (6)	−0.0053 (7)
N2	0.0431 (7)	0.0537 (8)	0.0422 (7)	0.0079 (6)	0.0114 (5)	0.0024 (6)
C1	0.0653 (11)	0.0582 (11)	0.0492 (10)	−0.0021 (9)	0.0114 (8)	0.0046 (8)
C2	0.0406 (8)	0.0525 (10)	0.0417 (8)	−0.0032 (7)	0.0077 (6)	−0.0012 (7)
C3	0.0529 (9)	0.0559 (10)	0.0417 (9)	0.0035 (7)	0.0163 (7)	−0.0050 (7)
C4	0.0444 (8)	0.0481 (9)	0.0485 (9)	0.0042 (7)	0.0149 (7)	−0.0079 (7)
C5	0.0817 (13)	0.0620 (12)	0.0632 (11)	0.0230 (10)	0.0271 (10)	−0.0048 (9)
C6	0.0680 (12)	0.0741 (14)	0.0632 (12)	−0.0250 (10)	0.0074 (9)	−0.0086 (10)
C7	0.0474 (8)	0.0576 (10)	0.0356 (8)	−0.0083 (8)	0.0028 (6)	0.0009 (7)
C8	0.0323 (7)	0.0653 (11)	0.0451 (9)	−0.0004 (7)	0.0081 (6)	0.0028 (7)
C9	0.0368 (7)	0.0515 (9)	0.0370 (8)	0.0066 (6)	0.0113 (6)	0.0068 (6)
C10	0.0443 (8)	0.0596 (11)	0.0674 (11)	0.0127 (7)	0.0233 (8)	0.0043 (8)
C11	0.0517 (10)	0.0963 (16)	0.0527 (11)	−0.0182 (10)	0.0053 (8)	−0.0141 (10)
C12	0.0449 (9)	0.0990 (16)	0.0484 (10)	0.0056 (9)	0.0017 (8)	0.0119 (10)
C13	0.0529 (10)	0.0562 (11)	0.0935 (14)	−0.0131 (9)	0.0175 (10)	−0.0120 (10)
C14	0.0375 (8)	0.0825 (14)	0.0785 (13)	−0.0044 (8)	0.0194 (8)	−0.0032 (11)
C15	0.0540 (10)	0.0724 (12)	0.0452 (9)	0.0086 (8)	0.0183 (8)	0.0043 (8)
C16	0.0905 (14)	0.0552 (11)	0.0627 (11)	0.0198 (10)	0.0256 (10)	0.0137 (9)

### Bis(acetylacetonato-κ2O,O')(N,N,N',N'-tetramethylethylenediamine-κ2N,N')zinc(II) (3) Geometric parameters (Å, º)

**Table:**

Zn—O1	2.0771 (12)	C6—H9	0.9800
Zn—O2	2.0611 (11)	C6—H10	0.9800
Zn—O3	2.0645 (11)	C6—C7	1.508 (2)
Zn—O4	2.0607 (10)	C7—C8	1.391 (3)
Zn—N1	2.2722 (13)	C8—H11	0.9500
Zn—N2	2.2533 (13)	C8—C9	1.393 (2)
O1—C2	1.2509 (19)	C9—C10	1.507 (2)
O2—C4	1.2578 (19)	C10—H12	0.9800
O3—C7	1.2619 (19)	C10—H13	0.9800
O4—C9	1.2626 (17)	C10—H14	0.9800
N1—C11	1.467 (2)	C11—H15	0.9900
N1—C13	1.469 (2)	C11—H16	0.9900
N1—C14	1.473 (2)	C11—C12	1.495 (3)
N2—C12	1.476 (2)	C12—H17	0.9900
N2—C15	1.470 (2)	C12—H18	0.9900
N2—C16	1.468 (2)	C13—H19	0.9800
C1—H1	0.9800	C13—H20	0.9800
C1—H2	0.9800	C13—H21	0.9800
C1—H3	0.9800	C14—H22	0.9800
C1—C2	1.512 (2)	C14—H23	0.9800
C2—C3	1.390 (2)	C14—H24	0.9800
C3—H4	0.9500	C15—H25	0.9800
C3—C4	1.392 (2)	C15—H26	0.9800
C4—C5	1.518 (2)	C15—H27	0.9800
C5—H5	0.9800	C16—H28	0.9800
C5—H6	0.9800	C16—H29	0.9800
C5—H7	0.9800	C16—H30	0.9800
C6—H8	0.9800		
			
O1—Zn—O2	87.50 (4)	C7—C6—H8	109.5
O1—Zn—O3	101.58 (5)	C7—C6—H9	109.5
O1—Zn—O4	88.49 (4)	C7—C6—H10	109.5
O1—Zn—N1	89.28 (5)	O3—C7—C6	115.59 (16)
O1—Zn—N2	168.94 (5)	O3—C7—C8	125.65 (15)
O2—Zn—O3	90.18 (5)	C8—C7—C6	118.76 (16)
O2—Zn—O4	175.16 (4)	C7—C8—H11	116.9
O2—Zn—N1	93.76 (5)	C7—C8—C9	126.12 (15)
O2—Zn—N2	89.57 (5)	C9—C8—H11	116.9
O3—Zn—O4	87.96 (4)	O4—C9—C8	125.48 (15)
O3—Zn—N1	168.61 (5)	O4—C9—C10	115.84 (15)
O3—Zn—N2	89.09 (5)	C8—C9—C10	118.67 (13)
O4—Zn—N1	88.92 (5)	C9—C10—H12	109.5
O4—Zn—N2	94.86 (5)	C9—C10—H13	109.5
N1—Zn—N2	80.27 (5)	C9—C10—H14	109.5
C2—O1—Zn	127.18 (11)	H12—C10—H13	109.5
C4—O2—Zn	126.86 (11)	H12—C10—H14	109.5
C7—O3—Zn	127.15 (11)	H13—C10—H14	109.5
C9—O4—Zn	127.15 (10)	N1—C11—H15	109.3
C11—N1—Zn	105.16 (10)	N1—C11—H16	109.3
C11—N1—C13	110.51 (16)	N1—C11—C12	111.53 (16)
C11—N1—C14	110.95 (15)	H15—C11—H16	108.0
C13—N1—Zn	111.23 (10)	C12—C11—H15	109.3
C13—N1—C14	107.86 (15)	C12—C11—H16	109.3
C14—N1—Zn	111.17 (11)	N2—C12—C11	111.49 (15)
C12—N2—Zn	105.59 (11)	N2—C12—H17	109.3
C15—N2—Zn	111.69 (10)	N2—C12—H18	109.3
C15—N2—C12	110.50 (15)	C11—C12—H17	109.3
C16—N2—Zn	111.08 (11)	C11—C12—H18	109.3
C16—N2—C12	110.16 (14)	H17—C12—H18	108.0
C16—N2—C15	107.84 (15)	N1—C13—H19	109.5
H1—C1—H2	109.5	N1—C13—H20	109.5
H1—C1—H3	109.5	N1—C13—H21	109.5
H2—C1—H3	109.5	H19—C13—H20	109.5
C2—C1—H1	109.5	H19—C13—H21	109.5
C2—C1—H2	109.5	H20—C13—H21	109.5
C2—C1—H3	109.5	N1—C14—H22	109.5
O1—C2—C1	116.12 (15)	N1—C14—H23	109.5
O1—C2—C3	126.06 (16)	N1—C14—H24	109.5
C3—C2—C1	117.82 (15)	H22—C14—H23	109.5
C2—C3—H4	117.4	H22—C14—H24	109.5
C2—C3—C4	125.30 (15)	H23—C14—H24	109.5
C4—C3—H4	117.4	N2—C15—H25	109.5
O2—C4—C3	126.43 (15)	N2—C15—H26	109.5
O2—C4—C5	115.53 (15)	N2—C15—H27	109.5
C3—C4—C5	118.02 (15)	H25—C15—H26	109.5
C4—C5—H5	109.5	H25—C15—H27	109.5
C4—C5—H6	109.5	H26—C15—H27	109.5
C4—C5—H7	109.5	N2—C16—H28	109.5
H5—C5—H6	109.5	N2—C16—H29	109.5
H5—C5—H7	109.5	N2—C16—H30	109.5
H6—C5—H7	109.5	H28—C16—H29	109.5
H8—C6—H9	109.5	H28—C16—H30	109.5
H8—C6—H10	109.5	H29—C16—H30	109.5
H9—C6—H10	109.5		

## References

[bb1] Albrecht, R., Liebing, P., Morgenstern, U., Wagner, C. & Merzweiler, K. (2019). *Z. Naturforsch. Teil B*, **74**, 233–240.

[bb2] Barreca, D., Carraro, G., Devi, A., Fois, E., Gasparotto, A., Seraglia, R., Maccato, C., Sada, C., Tabacchi, G., Tondello, E., Venzo, A. & Winter, M. (2012). *Dalton Trans.* **41**, 149–155. 10.1039/c1dt11342a 22048471

[bb3] Brahma, S., Sachin, H. P., Shivashankar, S. A., Narasimhamurthy, T. & Rathore, R. S. (2008). *Acta Cryst.* C**64**, m140–m143. 10.1107/S0108270108003818 18322327

[bb4] Brandenburg, K. (2019). *DIAMOND*. Crystal Impact GbR, Bonn, Germany.

[bb5] Dickman, M. H. (1998). *Acta Cryst.* C54 IUC9800048.

[bb6] Dolomanov, O. V., Bourhis, L. J., Gildea, R. J., Howard, J. A. K. & Puschmann, H. (2009). *J. Appl. Cryst.* **42**, 339–341.

[bb7] Gorkum, R. van, Buda, F., Kooijman, H., Spek, A. L., Bouwman, E. & Reedijk, J. (2005). *Eur. J. Inorg. Chem.* pp. 2255–2261.

[bb8] Groom, C. R., Bruno, I. J., Lightfoot, M. P. & Ward, S. C. (2016). *Acta Cryst.* B**72**, 171–179. 10.1107/S2052520616003954 PMC4822653 27048719

[bb9] Halbach, R. L., Nocton, G. & Andersen, R. A. (2012). *Dalton Trans.* **41**, 8809–8812. 10.1039/c2dt30810j 22706664

[bb10] Harbach, P., Lerner, H.-W. & Bolte, M. (2003). *Acta Cryst.* E**59**, m724–m725.

[bb11] Kaschube, W., Pörschke, K. R. & Wilke, G. J. (1988). *J. Organomet. Chem.* **355**, 525–532.

[bb12] Ma, Y. M., Reardon, D., Gambarotta, S., Yap, G., Zahalka, H. & Lemay, C. (1999). *Organometallics*, **18**, 2773–2781.

[bb13] Malandrino, G., Toro, R. G., Catalano, M. R., Fragalà, M. E., Rossi, P. & Paoli, P. (2012). *Eur. J. Inorg. Chem.* pp.1021–1024.

[bb14] Montgomery, H. & Lingafelter, E. C. (1968). *Acta Cryst.* B**24**, 1127–1128.

[bb15] Nelkenbaum, E., Kapon, M. & Eisen, M. S. (2005). *Organometallics*, **24**, 2645–2659.

[bb16] Ni, J., Yan, H., Wang, A., Yang, Y., Stern, C. L., Metz, A. W., Jin, S., Wang, L., Marks, T. J., Ireland, J. R. & Kannewurf, C. R. (2005). *J. Am. Chem. Soc.* **127**, 5613–5624. 10.1021/ja044643g 15826201

[bb17] Novitchi, G., Jiang, S., Shova, S., Rida, F., Hlavička, I., Orlita, M., Wernsdorfer, W., Hamze, R., Martins, C., Suaud, N., Guihéry, N., Barra, A.-L. & Train, C. (2017). *Inorg. Chem.* **56**, 14809–14822. 10.1021/acs.inorgchem.7b01861 29181984

[bb18] Pasko, S., Hubert-Pfalzgraf, L. G., Abrutis, A. & Vaissermann, J. (2004). *Polyhedron*, **23**, 735–741.

[bb19] Sheldrick, G. M. (2015*a*). *Acta Cryst.* A**71**, 3–8.

[bb20] Sheldrick, G. M. (2015*b*). *Acta Cryst.* C**71**, 3–8.

[bb21] Spek, A. L. (2009). *Acta Cryst.* D**65**, 148–155. 10.1107/S090744490804362X PMC2631630 19171970

[bb22] Stephens, F. S. (1977). *Acta Cryst.* B**33**, 3492–3495.

[bb23] Stoe & Cie (2016). *X-AREA.* Stoe & Cie, Darmstadt, Germany.

[bb24] Trimmel, G., Lembacher, C., Kickelbick, G. & Schubert, U. (2002). *New J. Chem.* **26**, 759–765.

[bb25] Tsodikov, M. V., Bukhtenko, O. V., Ellert, O. G., Petrunenko, I. A., Antsyshkina, A. S., Sadikov, G. G., Maksimov, Y. V., Titov, Y. V. & Novotortsev, V. M. (1995). *Russ. Chem. Bull.* **44**, 1396–1400.

[bb26] Zeller, A., Herdtweck, E. & Strassner, Th. (2004). *Inorg. Chem. Commun.* **7**, 296–301.

